# Co-loaded lapatinib/PAB by ferritin nanoparticles eliminated ECM-detached cluster cells via modulating EGFR in triple-negative breast cancer

**DOI:** 10.1038/s41419-022-05007-0

**Published:** 2022-06-20

**Authors:** Xinghan Wu, Huan Sheng, Liping Zhao, Mingxia Jiang, Han Lou, Yue Miao, Ni Cheng, Weifen Zhang, Dejun Ding, Wentong Li

**Affiliations:** 1 Department of Pathology, Weifang Medical University, Weifang, 261053 Shandong China; 2 College of Pharmacy, Weifang Medical University, Weifang, 261053 Shandong China; 3 School of Clinical Medicine, Weifang Medical University, Weifang, 261053 Shandong China; 4 Department of Oncology, The Second Affiliated Hospital of Shandong First Medical University, Taian, 271000 Shandong China; 5 Collaborative Innovation Center for Target Drug Delivery System, Weifang Medical University, Weifang, 261053 Shandong China

**Keywords:** Breast cancer, Cancer stem cells, Cancer therapy, Drug delivery

## Abstract

Cancer stem cell (CSC) cluster of triple-negative breast cancer (TNBC) is suggested to be responsible for therapy resistance, metastatic process and cancer recurrence, yet the sensitivity of CSC clusters of TNBC to ferroptosis remains elusive in a great measure. Current research revealed that epidermal growth factor receptor (EGFR) reinforced CD44-mediated TNBC cell clustering, whether blockade of EGFR has synergistic effects on erastin-induced tumor inhibition of CSC clusters is still poorly understood. Here, we found that fraction of CD24^low^CD44^high^ cells and size of tumor spheres clearly decreased following EGFR inhibition in TNBC cells. Inhibition of EGFR promoted expression of LC3B-II via YAP/mTOR signaling pathway, indicating that EGFR-mediated autophagy which contributed to ferroptosis. In order to further verify the protective effects of EGFR on ferroptosis induced by small molecules in TNBC cells, pseudolaric acid B (PAB) which led to ferroptosis of malignant cells was selected. In our experiment, lapatinib and PAB cotreatment inhibited TNBC cells viability and restrained formation of tumor spheres, accompanied with a high level of intracellular ROS. To target delivery lapatinib and PAB to TNBC cells, lapatinib/PAB@Ferritin (L/P@Ferritin) nanoparticles were prepared; results of in vitro and in vivo showed a higher tumor suppression efficiency of L/P@Ferritin, highlighting that it might provide a new perspective for treatment of CSC clusters of TNBC.

## Introduction

Triple-negative breast cancer (TNBC) tends to detach from extracellular matrix (ECM), resulting in increased risk of distant metastasis [1]. Ferroptosis, an iron-dependent programmed cell death, defined by overwhelming intracellular accumulation of lethal lipid reactive oxygen species (ROS) [2]. Mounting evidence specified that TNBC cells were sensitive to oxidative stress and ferroptosis [3]; therefore, ferroptosis might be a potential therapeutic strategy for TNBC.

Polyclonal metastasis of TNBC engendered by circulating tumor cell (CTC) clusters are driven by assemblage of cancer stem cells (CSC). The ECM-detached cells, when displaying CTC clusters, are responsible for intrinsic resistance to chemotherapy [4]; whether that ECM-detached CTC clusters of TNBC are stand up to ferroptosis is unclear, and how to kill the clusters of ECM-detached cell clusters is still a challenge to overcome metastasized breast cancer.

Flourishing epidermal growth factor receptor (EGFR) is considerably conjoined with stemness, drug resistance and metastasis in various cancers, in particular TNBC [5]. EGFR was upregulated in the CTC fraction in breast xenograft model and head and neck squamous cell carcinoma patients with locally advanced disease [6], EGFR promoted TNBC cell clustering and blockade of EGFR successfully abolished tumor cell cluster formation [7]. Ferroptosis is proposed as a style of autophagy-associated cell death [8], recent testimonies confirmed that pseudolaric acid B (PAB) could achieve antitumor effects and induce ferroptosis by inducing autophagy [9] and upregulating transferrin receptor (TfR) [10]. A growing number of studies indicated that autophagy was hampered by activating EGFR signaling pathway to survive under stresses [11]. Whether that EGFR inhibition impedes formation of ECM-detached cell clusters, enhances autophagy and achieves the purpose of tumor inhibition via ferroptosis remains to be explored.

Here, we demonstrated that inhibition of EGFR signaling pathway significantly suppressed cell viability of TNBC cells and reduced fraction of CSCs with intracellular enhancement of lipid peroxidation when TNBC cells exposed to erastin. Further, we prepared a ferritin nanoparticle (L/P@Ferritin) co-loaded with lapatinib/PAB to achieve the purpose of better delivery of EGFR inhibitors and ferroptosis inducers to weaken stemness, induce autophagy and ferroptosis. The nanoparticle was substantiated with exceptional selective tumoricidal action through inducing ferroptosis on TNBC cells in vitro and in vivo. In brief, our research certified that L/P@Ferritin nanoparticle provided a new perspective for the treatments of TNBC to induce ferroptosis by modulating EGFR signaling pathway.

## Materials and methods

### Cell culture and DNA transfection

Immortalized human mammary epithelial cell MCF-10A and human TNBC cell lines MDA-MB-231, MDA-MB-453, and MDA-MB-468 were obtained from the American type culture collection (ATCC, USA). Cells were maintained in RPMI-1640 or DMEM medium (Gibco, NY, USA) supplemented with 10% fetal bovine serum (Evergreen, Beijing, China) at 37 °C in a humidified atmosphere of 5% CO_2_. Low adhesion 24-well plate (Corning Costar, NY, USA) was used to carry out spheroid forming assays. PLKO.1-Puro-TRC-shEGFR (Tsingke Biotechnology, Beijing, China), pHAGE-EGFR (116731, Addgene) constructs were transfected into 293 T cells using the Lipofectamine 2000 reagent (Invitrogen, Carlsbad, CA) to package lentivirus and TNBC cell lines were infected with the packaged lentivirus according to manufacturer’s instruction.

### Cell viability assay and EdU proliferation assay

5 × 10^3^ TNBC cells were planted in a 96-well plate per well at 37 °C for 48 h. Then, 5 mg/mL 3-(4,5-dimethyl-2-thiazolyl)-2,5-diphenyltetrazolium bromide (MTT) solution (10 μL/well) was added and incubated at 37 °C for 4 h. 100 μL dimethyl sulfoxide was used to dissolve crystallization. Absorbance at the wavelength of 490 nm was detected by automatic enzyme marker (Multiskan GO, Thermo, USA). For EdU assay, 5 × 10^4^ TNBC cells were seeded in 24-well plate per well; cell Proliferation Kit (Beyotime Biotechnology, Nanjing, China) was applied to detect cell proliferation by fluorescence microscope (X-73; Olympus, Tokyo, Japan).

### Wound healing and colony formation assay

In wound healing assay, 2 × 10^5^ cells were seeded in 6-well plate per well for 24 h, a straight line was scratched across the surface of cells, migration distance was detected at 0 h, 24 h, and 48 h. For colony formation, 3 × 10^2^ cells were seeded in 6-well plate for 14 days, after being fixed with 4% paraformaldehyde, cells were stained with 0.1% crystal violet (MedChemExpress). Colonies >50 cells were identified and counted.

### Flow cytometry (FCM)

For cell cycle analysis, cells were fixed with 70% cold ethanol and kept at 4 °C overnight. Cells were labeled with propidium iodide (Solarbio, Beijing, China). BD FACSCanto II flow cytometry (C6plus; BD, CA, USA) was used to analyze cell cycle distribution. 7-ADD and Annexin V-FITC double staining kit (BD, CA, USA) was applied to identify ferroptosis. To detect CD44 and CD24, cells were incubated with PE anti-human CD24 antibody (311105; BioLegend, CA, USA) and FITC anti-mouse/human CD44 antibody (103005; BioLegend, CA, USA) in 2% FBS/HBSS in the darkness for 20 min, cells were gauged by FACS Calibur Flow Cytometer (Aria 3; BD, CA, USA).

### Assessment of ROS, lipid peroxidation, and ferrous iron

Cells were cultured at 2 × 10^5^ cells/well in 6-well plate, ROS assay kit (Beyotime Biotech, Nanjing, China) was resorted to measuring generation of ROS by fluorescence microscopy and FCM. For lipid peroxidation, cells were incubated with 5 μM C11-BODIPY (D3861; Thermo Fisher, OR, USA), the signal were acquired by confocal laser scanning microscopy (CLSM, Leica TCS SP8; Leica Microsystems, Wetzlar, Germany) and FCM. Cells were plated in 6-well at 2 × 10^5^ cells/well, an iron colorimetric assay kit (Abcam, Cambridge, UK) was used to detect intracellular ferrous iron; absorbance at the wavelength of 593 nm was defined.

### Measurement of intracellular glutathione (GSH) and malondialdehyde (MDA)

5 × 10^4^ cells/well were inoculated in 6-well plates. The levels of MDA and GSH were detected with MDA assay kit (Jiancheng Bioengineering, Nanjing, China) and GSH assay kit (Beyotime, Nanjing, China).

### Transmission electron microscopy (TEM)

To observe the alteration of cell ultrastructural features, cells were inoculated in 10 cm^2^ dishes and exposed to drugs for 48 h. After that, cells were collected and fixed with 3% glutaraldehyde. Samples were pretreated according to standard procedures, then images were acquired with TEM (HT-7700, Hitachi, Tokyo, Japan).

### Mitochondrial membrane potential (ΔΨM) assay

2 × 10^5^ cells/well of cells were seeded in 6-well plate, cells were labeled with tetrachloro-tetraethyl benzimidazol carbocyanine iodide 1 (JC-1) with mitochondrial membrane potential assay kit with JC-1 (Beyotime Biotech, Nanjing, China) following the manufacture’s protocol. The results were observed with fluorescence microscopy.

### Western blot

Total proteins were extracted from cells with RIPA buffer (Solarbio, Beijing, China), proteins were separated by SDS-polyacrylamide gels, then transferred to polyvinylidene difluoride membranes. The membranes were incubated with primary antibodies directed against LC3B (L7543, Sigma-Aldrich, MO, USA), Atg7 (#8558, Cell Signaling Technology, MA, USA), FTH1 (#4393, Cell Signaling Technology), EGFR (A11351, ABclonal, Wuhan, China), N-cadherin (A19083, ABclonal), YAP (A1002, ABclonal), p-YAP (AP0489, ABclonal), mTOR (A2445, ABclonal), p-mTOR (AP0115, ABclonal), TfR (14-0719-82, Invitrogen, CA, USA), β-actin (TA-09, Zsgb Bio, Beijing, China) overnight at 4 °C. Secondary antibodies conjugated with HRP (Proteintech, Wuhan, China) were used and blots were presented with FluorChem Q Imager (ProteinSimple, CA, USA) after incubating with ECL (Beyotime Biotech, Nanjing, China).

### Immunofluorescence microscopy

5 × 10^4^ cells/well cells were cultivated and fixed by 4% paraformaldehyde, followed by permeabilization in 0.1% Triton-X 100. Primary antibodies and fluorescein-conjugated secondary antibodies were incubated with samples, respectively. Results were visualized using CLSM.

### Preparation of L/P@Ferritin nanodrug

Lapatinib and PAB were dissolved in oil phase of dichloromethane, ferritin (Sigma-Aldrich, MO, USA) was dissolved in H_2_O to 1 mg/mL as the aqueous phase. The emulsion in which oil phase and aqueous phase were mixed at the ratio of 1:1 was sonicated in a mechanical sonicator (Scientz, Ningbo, China) for 1 h, the emulsion was dialyzed, nano-drugs were acquired through lyophilization.

### Characterization and stability assay

TEM was performed to reveal the morphological features of the nanoparticles by putting L/P@Ferritin onto a carbon-coated grid with holes. Dynamic light scattering (DLS) and zeta potential (Zetasizer Nano system Malvern Instruments, Malvern, UK) were performed to assess size distribution and electrical stability. High-performance liquid chromatography (HPLC; Waters Alliance e2695, NY, USA) equipped with a 2489 UV/Vis detector was used to evaluate drug encapsulation and loading efficiency. The efficiencies of drug loading and encapsulation were determined as our previous report [12]. To investigate the stability, L/P@Ferritin was dissolved in water, PBS (pH 7.4) and cell culture medium; then the hydrodynamic radius of nanodrug were evaluated by DLS.

### Hemolysis assay and In vitro release of lapatinib and PAB by L/P@Ferritin

Hemolysis assay was carried out to evaluate biosafety of L/P@Ferritin. L/P@Ferritin was dissolved in 2 mL PBS and wrapped in a pretreated dialysis membrane, then dialyzed in 48 mL PBS at a pH of 6.5 or 7.4 at 37 °C. Lapatinib and PAB released in the supernatant were evaluated with HPLC.

### In vivo experiments

Animal experiments were approved by the Ethics Committee of Weifang Medical University (2020SDL117). 5 × 10^6^ MDA-MB-231 cells were subcutaneously injected into the right hind limb of BALB/c nude female mice (age: 4 weeks; weight: 12–17 g; Vital River, Beijing, China). When tumor volume reached to 70–130 mm^3^, 30 mice were randomly divided into 6 groups: control (PBS), lapatinib (15 mg/kg), PAB (10 mg/kg), combination (lapatinib/PAB), vehicle (ferritin) and nanoparticle (L/P@Ferritin). Each group was treated with drugs by intraperitoneal injection every 2 days for seven times. Tumor sizes were evaluated and calculated by the following formula: 0.5 × length × width^2^. The mice were typically euthanized by CO_2_, tumors, and organs were collected for immunofluorescence and HE stain.

To generate lung metastases model, 1 × 10^6^ MDA-MB-231-luc cells were injected intravenously into NOD-SCID immunodeficient mice (female, age: 4 weeks; Vital River, Beijing, China). 12 mice were randomly divided into three groups: control (PBS), combination (lapatinib/PAB) and nanoparticle (L/P@Ferritin). Each group was treated with drugs by tail vein injection every 3 days for seven times after inoculation 14 days. Lung metastasis was evaluated using the PE IVIS Spectrum (PerkinElmer, MA, USA).

### Statistical analysis

Results were shown as means ± SEM, each experiment was repeated for three times. The statistical analyses were performed by one-way analysis of variance and student’s *t*-test as indicated in the text using GraphPad Prism 8. When the experiment that has more than two groups, followed by Bonferroni/Dunn post hoc comparison of means with correction for multiple comparisons. *P < 0.05 was considered statistically significant. **P < 0.01 were considered highly statistically significant.

## Results

### EGFR was associated with ECM-detached characteristics and CSCs ferroptosis resistance

Detachment of malignant cells from ECM is a pro-ferroptotic stress [13], Prominin2 facilitates ferroptosis resistance in ECM-detached condition [14]. We reasoned that ECM-detached cells survive detached conditions by upregulating other protective genes. Here, RNA-sequencing data were used to compare mRNA expressions in adherent and ECM-detached MCF-10A (GSE115059), there were 50 and 72 genes with substantially augmented or declined expression, respectively (Fig. S1A); notably, EGFR was among the upregulated genes upon ECM-detachment. Then we import those differential genes into the DAVID database (https://david.ncifcrf.gov/) for Gene Ontology (GO) and Kyoto Encyclopedia of Genes and Genomes (KEGG) analysis. We found that those differential genes mainly associated with plasma membrane, integral component of plasma membrane, lipid metabolism process and cell adhesion (Fig. S1B). The top KEGG pathways enriched for the differential genes in adherent and ECM-detached were mainly related to metabolism pathways, ECM-receptor interaction (Fig. S1C). Based on GEO cancer dataset (GSE115059, GSE62931), gene set enrichment analysis (GSEA) uncovered robust interactions between CSCs gene category and ferroptosis in TNBC patients (Fig. S1D). Moreover, TNBC patients with elevated EGFR were correlated with poor prognosis based on the kmplot dataset (https://kmplot.com/analysis/index.php?p=background, Fig. S1E).

### Overexpression of EGFR promoted resistance to ferroptotic cell death

**Fig. 1:**
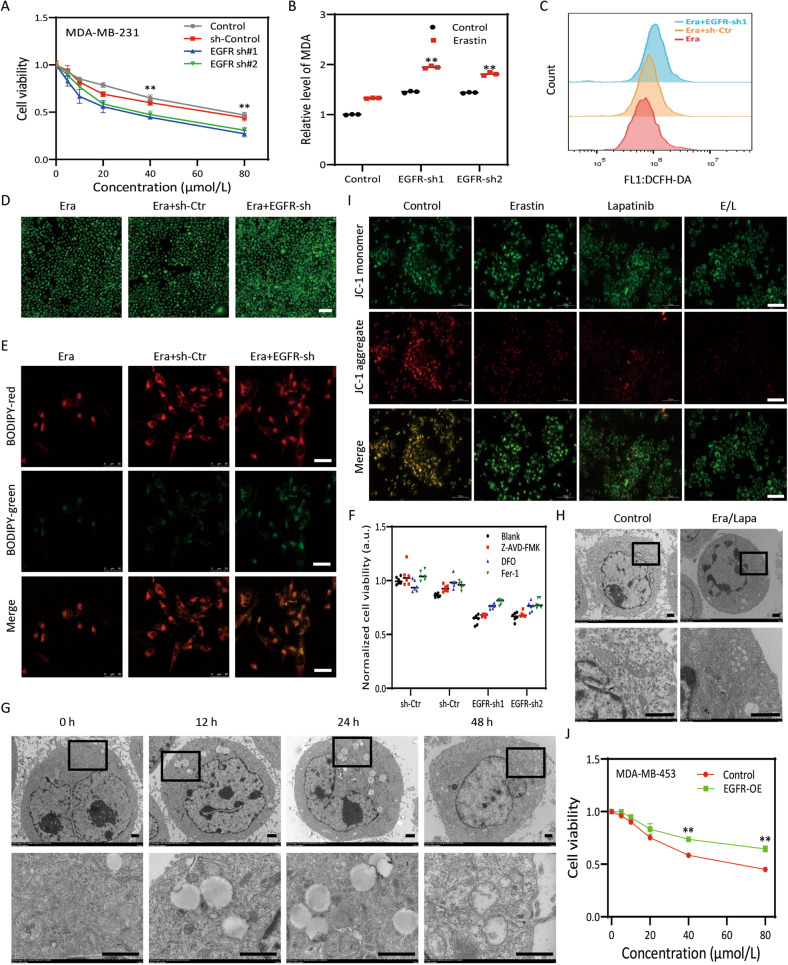
Overexpression of EGFR promoted resistance to ferroptotic cell death. **A** Effects of knockdown of EGFR on erastin-induced growth inhibition in MDA-MB-231 cells. **B** Analysis of intracellular MDA in MDA-MB-231 cells. **C**, **D** ROS generation in MDA-MB-231 cells detected by FCM and fluorescence microscopy; scale bars represent 100 μm. **E** Lipid ROS generation in MDA-MB-231 cells detected by laser confocal; scale bar, 50 μm. **F** MDA-MB-231 cells treatment with Fer-1, DFO and Z-VAD-FMK mitigated erastin/sh-EGFR induced decrease in cell viability by MTT assay. **G**, **H** Shrunken mitochondria with increased membrane density when treated with erastin and/or lapatinib in MDA-MB-231 cells; scale bars represent 1 μm. **I** Erastin/lapatinib increased the ratio of JC-1 monomers to JC-1 aggregates in MDA-MB-231 cells; scale bars represent 100 μm. **J** Effects of overexpression EGFR on erastin-induced growth inhibition in MDA-MB-453 cells. *P < 0.05, **P < 0.01.

As EGFR is associated with ECM-detached characteristics and CSCs, the role of EGFR in ferroptosis resistance needs to be characterized. Reduction of EGFR by shRNA (Figure S2A) or EGFR inhibitor augmented erastin-induced growth inhibition in MDA-MB-231 and MDA-MB-468 cell lines (Fig. 1A, S2B), associated with increased intracellular MDA (Fig. 1B), ROS (Figs. 1C, 1D) and lipid ROS (Fig. 1E, S2C). Moreover, deferoxamine (DFO, ferroptosis inhibitor) and ferrostatin-1 (Fer-1, ferroptosis inhibitor), but not Z-VAD-FMK (pan caspase inhibitor), prevented erastin-induced growth inhibition in EGFR-silenced MDA-MB-231 and MDA-MB-468 cells (Fig. 1F, S2D). Inhibition of EGFR by lapatinib similarly strengthened erastin-induced growth inhibition (Fig. S2E), indicating that blockade of EGFR definitely consolidated erastin-induced ferroptosis in TNBC cells. Erastin resulted in accumulation of fatty droplets, mitochondria disrupted cristae and shrunken mitochondria with increased membrane density at 12 h in TNBC cells (Fig. 1G), indicating that lipid metabolism disturbance occurred at the early stage of ferroptosis which was in compliance with previous report [15]; however, in our research, only some small to medium lipid droplets could be found compared to large liposphere located in the center of lesion cells [15]. At 48 h, MDA-MB-231 cells showed mitochondria swell, rupture of mitochondrial membrane, condensed chromosome at the nuclear periphery (Fig. 1G), more serious structural aberrations, such as cell membrane rupture, appeared when lapatinib and erastin was used (Fig. 1H). JC-1 assay displayed that lapatinib/erastin contributed to prominent reduction of red fluorescence and elevation of the green fluorescence in MDA-MB-231 cells (Fig. 1I), highlighting that lapatinib/erastin induced distinct mitochondrial depolarization. Predictably, aberrant EGFR in MDA-MB-453 cells (a TNBC cell line possess a mild EGFR expression) responsible for rescue of erastin-induced growth inhibition confirmed by MTT assay (Fig. 1J, S2F).

### Blockage of EGFR sensitized TNBC to ferroptosis via reducing stemness

**Fig. 2:**
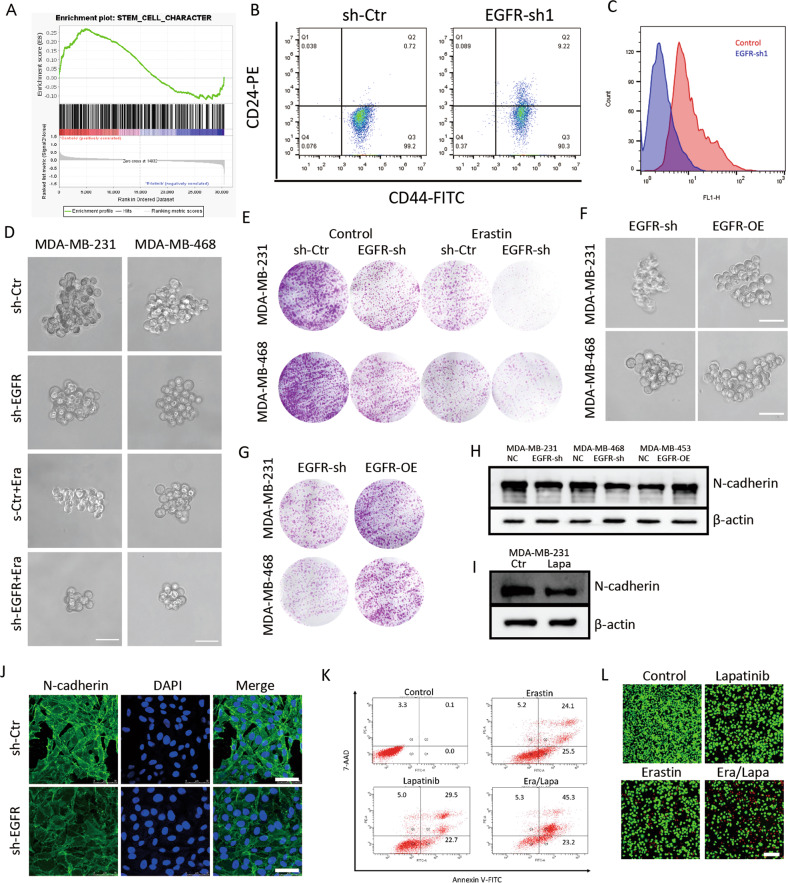
Blockage of EGFR sensitized TNBC to ferroptosis via weakening stemness. **A** Weak stemness was associated with EGFR blockade based on GSEA. **B** Proportion of CD44^high^CD24^low^ was detected by FCM in sh-EGFR/MDA-MB-231 cells. **C** ALDH subpopulation analysis was detected by FCM in sh-EGFR/MDA-MB-231 cells. **D** Formation of three-dimensional spheroids of MDA-MB-231 cells and MDA-MB-468 cells when silenced EGFR and/or treated with erastin; scale bars represent 50 μm. **E** Results of colony formation assay of MDA-MB-231 cells and MDA-MB-468 cells when silenced EGFR and/or treated with erastin. **F** Overexpression EGFR rescued the ability of three-dimensional spheroids formation in MDA-MB-231 cells and MDA-MB-468 cells; scale bars represent 50 μm. **G** Results of the colony formation assay of MDA-MB-231 cells and MDA-MB-468 cells when overexpression EGFR. **H** Protein levels of N-cadherin in TNBC cells deteched by western blot. **I** Expression of N-cadherin after treated with lapatinib in MDA-MB-231 cells. **J** N-cadherin was visualized by confocal microscopy in sh-EGFR/MDA-MB-231 cells; scale bars represent 50 μm. **K** Cell ferroptosis displayed by FCM of MDA-MB-231 cells treated with erastin and/or lapatinib. **L** Results of MDA-MB-231 cells live/dead cell stain after treated with erastin and/or lapatinib; scale bars represent 200 μm.

Using the GEO database (GSE45891), GSEA was carried out based on EGFR expression and we found differential genes upon EGFR blockade associated with weaken stemness (Fig. 2A). Here, lapatinib was used to further investigate whether EGFR is related to stemness of TNBC cells. The fraction of CD24^low^CD44^high^ cells clearly decreased upon EGFR silence in MDA-MB-231 (Fig. 2B); the profile of CD24^low^CD44^high^ cells was also considerably diminished following lapatinib intervention (Fig. S3A). Aldehyde dehydrogenase (ALDH) in both cells were significantly reduced also upon EGFR knockdown or lapatinib treatment (Fig. 2C, S3B). Knockdown or blockade of EGFR remarkably reduced the size of tumor spheres (Fig. 2D, S3C) and ability of clonal formation in both TNBC cell lines (Fig. 2E, S3D). Exogenous EGFR rescued impaired abilities of tumor spheres formation and colony formation mediated by sh-EGFR (Fig. 2F, G), and spheroid tumor cells were insensitive to erastin-induced ferroptosis (Fig. S3E). Given N-cadherin was enriched in tumorsphere cells and associated with cancer stem cell-like characteristics [16], expression of N-cadherin was revealed in this study. N-cadherin was reduced when EGFR was blockaded in TNBC cells (Fig. 2H, I and J); otherwise, overexpressed-EGFR upregulated N-cadherin in MDA-MB-453 cells (Fig. 2H), accompanied with enlarged size of tumor spheres (Fig. S3F); ADH-1 (MedChemExpress, Shanghai, China), a N-cadherin antagonist, repressed EGFR-mediated enlarged size of tumor spheres (Fig. S3F).

As failure to eradicate CSCs profoundly dedicated to drug resistance, we interrogated whether EGFR participated in ferroptotic sensitivity. MTT assay showed that erastin apparently inhibited cell viability of EGFR-silenced cell compared to parental MDA-MB-231 cell (Fig. 1A); on the contrary, MDA-MB-453 cells with exogenous of EGFR was insensitive to erastin-induced ferroptosis (Fig. 1J). Inhibition of EGFR further confirmed the same conclusion by FCM (Fig. 2K). Results of live/dead cell stain assay revealed that knockdown or blockade of EGFR enhanced erastin-induced antitumor effects (Fig. 2L, S3G). As shown by tumor spheroid formation assay and colony formation assay, silence of EGFR in MDA-MB-231 exhibited limited tumor initiating capacity when treated with erastin (Fig. 2D, E).

### EGFR-blockade sensitized ECM-detached tumor cells to ferroptosis

**Fig. 3:**
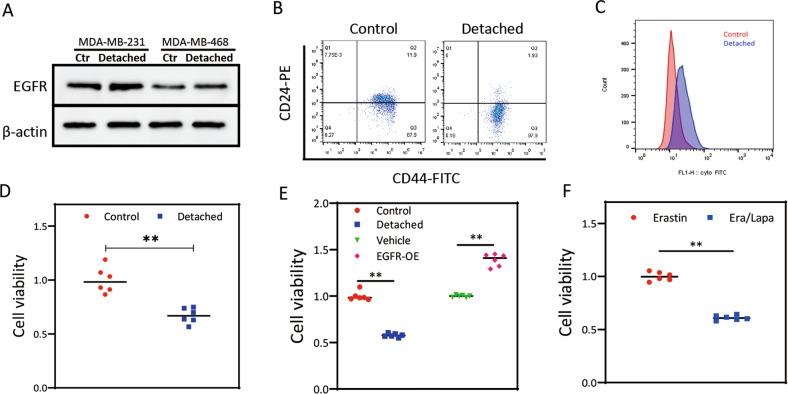
Sensitivity to ferroptosis in ECM-detached tumor cells with EGFR blockade. **A** Protein expression levels of EGFR in ECM-detached TNBC cells. **B** FCM analysis of CD44 and CD24 proportion in ECM-detached MDA-MB-231 cells. **C** ALDH subpopulation analysis in ECM-detached MDA-MB-231 cells detected by FCM. **D** Cell viability was detected under attached or detached condition in MDA-MB-231 cells. **E**, **F** Cell viabilities were induced by erastin with EGFR overexpression or lapatinib in MDA-MB-231 cells. **P < 0.01.

The aforementioned computational analyses revealed potential relevance between ECM-detachment and EGFR based on GSE115059 (Fig. S1A). To further investigate whether EGFR was related to ECM-detached characteristics in TNBC cells, we observed that ECM-detached MDA-MB-231 cells presented higher EGFR expression (Fig. 3A). Increasing evidence suggested that ECM-detachment promoted chemoresistance and expression of stemness markers in breast cancer cells [17]. Here, we found that ECM-detachment increased proportion of ALDH^+^ and CD44^high^CD24^low^ subsets (Fig. 3B, C). In addition, ECM-detached cells manifested a plentiful reduction in viability (Fig. 3D). ECM-detachment enhanced erastin-induced ferroptosis sensitivity; whereas, ECM-detached cells overexpressing EGFR were sufficient to relief erastin-induced ferroptosis (Fig. 3E). Next, we found lapatinib sensitized erastin-induced ferroptotic cell death in ECM-detached cells (Fig. 3F).

### EGFR inhibition promoted ferroptotic sensitivity via enhancing autophagy

**Fig. 4:**
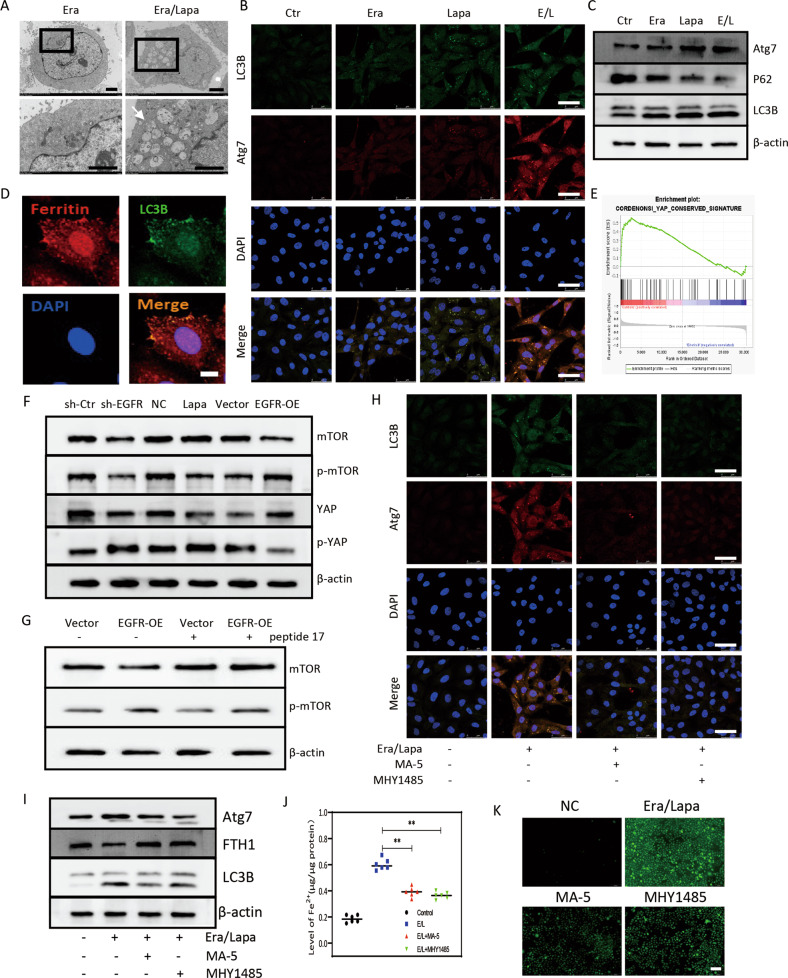
EGFR inhibition promoted ferroptotic sensitivity via enhancing autophagy. **A** TEM results showed ultrastructural features of autophagy in MDA-MB-231 cells when treated with erastin and/or lapatinib; scale bars represent 2 μm. **B** LC3B and Atg7 visualized by CLSM in MDA-MB-231 cells when treated with erastin and/or lapatinib, scale bars represent 50 μm. **C** Western blot analysis for LC3B, Atg7 and P62 in MDA-MB-231 cells when treated with erastin and/or lapatinib. **D** CLSM images of ferritin and LC3B in MDA-MB-231 cells when treated with erastin and lapatinib, scale bars represent 10 μm. **E** GSEA of YAP signaling pathway in EGFR-blockade cells. **F**, **G** p-mTOR and YAP expression in MDA-MB-231 cells transfected with sh-EGFR or EGFR-OE. **H** LC3 and Atg7 in MDA-MB-231 cells visualized by CLSM, scale bars represent 50 μm. **I** Western blot analysis for LC3B, Atg7 and FTH1 in MDA-MB-231 cells. **J** Intracellular ferrous iron in MDA-MB-231 cells. **K** ROS generation in MDA-MB-231 cells, scale bars represent 100 μm. **P < 0.01.

Fortified ferritinophagy degraded ferritin to increase intercellular iron and resulted in the activation of iron-dependent enzyme lipoxygenases during ferroptosis [18]. In this study, more autophagosomes (white arrows) were perceived in the cytoplasm of lapatinib/erastin-treated MDA-MB-231 cells compared to the erastin-treated cells (Fig. 4A). Inhibition of EGFR promoted erastin-induced expression of microtubule-associated protein light chain 3B I/II (LC3B-I/II), P62 and autophagy related protein 7 (Atg7) confirmed by immunofluorescence and western blot (Fig. 4B, C). To explore the relationship between canonical ferritin release and EGFR, we visualized subcellular allocation of ferritin in MDA-MB-231 incubated with 100 μg/mL ferric ammonium citrate (FAC), a punctate distribution of intracellular ferritin was observed (Fig. 4D). More ferritin was found to colocalize with LC3B in EGFR-inhibited TNBC cells treated with erastin (Fig. 4D), but not with cis-Golgi marker (Fig. S4A). Furthermore, we witnessed that ferritin partially colocalized with nuclear receptor coactivator 4 (NCOA4) (Fig. S4B).

As reported, Yes-associated protein (YAP) and mTOR were proved to be involved in autophagy processes [19–21], YAP signaling pathway (http://www.gsea-msigdb.org/gsea/msigdb/cards/CORDENONSI_YAP_CONSERVED_SIGNATURE.html) was associated EGFR blockade in a GEO dataset (GSE45891) based on GSEA analysis (Fig. 4E), thus we inferred that inhibition of EGFR might modulate autophagy and sensitize ferroptosis by controlling YAP/mTOR. Here, significantly reduced mTOR phosphorylation and decreased YAP were observed in MDA-MB-231 cells when EGFR was knockdown or inhibited by antagonist (Fig. 4F). On the contrary, overexpression of EGFR in MDA-MB-453 cells contributed to activation of p-mTOR and YAP (Fig. 4F); notably, peptide 17 (HY-P2244, MedChemExpress), a YAP inhibitor, exerted a significant attenuating effect on the activation of mTOR pathway caused by overexpression of EGFR (Fig. 4G). Next, we assessed the possible contribution of autophagy to the role of EGFR on erastin-induced ferroptosis, either activation of YAP by mitochonic acid 5 (MA-5, HY-111536, MedChemExpress) or mTOR by MHY1485 (HY-B0795, MedChemExpress) alleviated formation of lapatinib/erastin-mediated autophagosomes (Fig. 4H), reduced LC3B-II and Atg7 and rescued ferritin (Fig. 4I). Decreased intracellular levels of iron and ROS by activating mTOR or YAP indicated a downregulated autophagic capacity pathway that related to ferroptotic resistance of TNBC cells (Fig. 4J, K).

### PAB restrained cell viability and increased intracellular iron and ROS

**Fig. 5:**
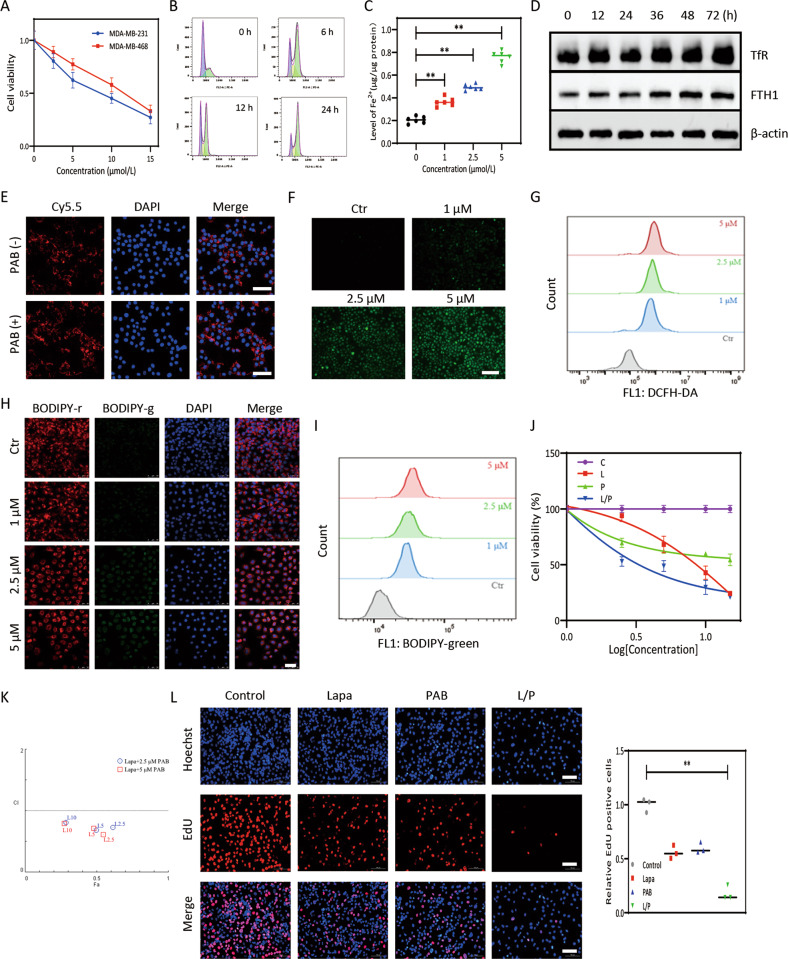
PAB inhibited cell viability and increased intracellular ferrous iron and ROS. **A** Growth inhibitory effect of PAB in MDA-MB-231 cells and MDA-MB-468 cells. **B** Flow cytometric analysis of cell cycle distribution by PAB in MDA-MB-231 cells. **C** PAB increased intracellular ferrous iron in MDA-MB-231 cells. **D** PAB increased expression of TfR and FTH in MDA-MB-231 cells. **E** PAB increased Cy5.5-ferritin uptake in MDA-MB-231 cells, scale bars represent 100 μm. **F** PAB increased intracellular ROS production in MDA-MB-231 cells; scale bars represent 200 μm. **G** Quantitative results of ROS via FCM in MDA-MB-231 cells. **H** PAB increased intracellular lipid ROS production in MDA-MB-231 cells; scale bars represent 50 μm. **I** Lipid peroxidation via FCM in MDA-MB-231 cells. **J** Lapatinib and PAB inhibited cell viability in MDA-MB-231 cells. **K** The CI (combined index) value was calculated between lapatinib and PAB by the Chou-Talalay. **L** Proliferative ability of lapatinib and/or PAB treated MDA-MB-231 cells displayed by EdU assay; scale bars represent 100 μm. **P < 0.01.

Accumulating evidence manifested that PAB was cytotoxicity to multiple tumor cells [22], here, the effects of PAB against human TNBC cells were queried. As shown in Fig. 5A, the viabilities of MDA-MB-231 and MDA-MB-468 cells were attenuated drastically by PAB in a dose-dependent manner. PAB evoked an evident reduction of cells in G0/G1 phase and a concomitant aggregation of cells arrested in G2/M phase in TNBC cells (Fig. 5B). Previous studies showed that PAB could upregulate expression of TfR [10], so we assayed the changes of intracellular ferrous irons after PAB treatment; compared to control group, ferrous iron was elevated apparently after being treated with PAB, and the increase was more considerable when PAB was elevated to 5.0 μmol/L (Fig. 5C). Both TfR and ferritin heavy chain 1(FTH1) were time-dependently augmented after treated with PAB in MDA-MB-231 cells were displayed by western blot (Fig. 5D). To prove whether PAB promoted uptake of ferritin in TNBC cells, results of cell uptake showed that co-incubation with PAB and Cy5.5-ferritin increased uptake of ferritin significantly in MDA-MB-231 (Fig. 5E). PAB engendered dose-dependent accumulation of cytosolic and lipid ROS confirmed by FCM and CLSM (Fig. 5F–I).

Results of MTT indicated that inhibitory effect of combined treatment with lapatinib and PAB was significantly enhanced compared to single-agent therapy (Fig. 5J). And it was validated that the best synergic effect was achieved when the ratio of lapatinib to PAB was 1:1 by Chou-Talalay method (Fig. 5K). EdU assay indicated that combined therapy has a stronger restraint on DNA synthesis (Fig. 5L). Wound healing assay showed a significant reduction in migration distance after co-incubation with lapatinib/PAB (Fig. S5A). As shown in Fig. S5B and S5C, ROS generation significantly increased by lapatinib/PAB which was rescued by ferroptosis inhibitor DFO or Fer-1. MDA-MB-231 cells treated with lapatinib/PAB showed a marked increased C11-BODIPY fluorescence signal (Fig. S5D). Generation of MDA was increased when cells were treated with lapatinib/PAB which could be markedly restrained in presence of DFO (Fig. S5E). Lapatinib/PAB significantly reduced intracellular GSH content which could be abrogated by application of Fer-1 or DFO (Fig. S5F).

### Synthesis and characterization of L/P@Ferritin nanodrug

**Fig. 6:**
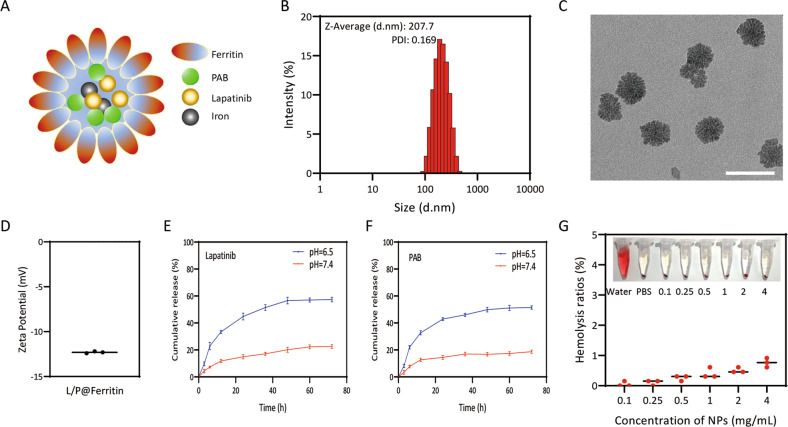
Synthesis and characterization of L/P@Ferritin nanodrug. **A** The schematic of synthesized L/P@Ferritin nanodrug by emulsification. **B**, **C** Size of L/P@Ferritin determined by DLS and TEM, scale bars represent 100 nm. **D** Zeta potential of L/P@Ferritin. **E** In vitro release of lapatinib from L/P@Ferritin. **F** In vitro release of PAB from L/P@Ferritin. **G** Hemolysis test of L/P@Ferritin nanodrug.

Due to poor solubility of lapatinib and PAB, we prepared co-loaded lapatinib/PAB ferritin nanoparticles (L/P@Ferritin) by emulsification to allow systemic administration on TNBC (Fig. 6A). The size distribution of L/P@Ferritin determined by DLS with an average particle size of 222.6 ± 11.2 nm (Fig. 6B). Results of TEM images showed that L/P@Ferritin had a sphere-like shape composed of many subunits and a uniform size of around 70 nm (Fig. 6C), the zeta potential of L/P@Ferritin was −12.3 ± 0.08 mV (Fig. 6D). The negative charge on the surface of L/P@Ferritin could effectively avoid phagocytosis by reticuloendothelial system [23]. There was no significant changes in particle size in different mediums and various temperatures for 3 days (Fig. S6A, S6B), while diameter was a slightly increase in the PBS and cell medium which may be caused by salts in the solutions (Fig. S6A), indicating that L/P@Ferritin has good stability. Drug-loading efficiency of L/P@Ferritin was analyzed by HPLC and the highest drug-loading ratios for lapatinib and PAB were 3.72% and 2.32%, the highest drug encapsulation efficiencies were 38.06% and 74.24%.

The release of lapatinib and PAB in L/P@Ferritin was investigated by dialysis in PBS (pH 7.4) for 72 h. As shown in Fig. 6E, both lapatinib and PAB were released faster in the first 24 h with 22% and 18% release. Furthermore, the cumulative release reached a platform at 48 h. Energy deficient contributed to acidic tumor microenvironment, in this research, 57% and 51% lapatinib and PAB were released under acidic conditions at 24 h (pH 6.5), revealing that L/P@Ferritin hydrolyze under acidic conditions and almost degrade (Fig. 6F), since ferritin could be disassembled by altering the buffer to acidic or basic [24]. Centrifugal RBC solution and hemolysis rate were shown in Fig. 6G, even 4 mg/mL L/P@Ferritin showed very low hemolytic toxicity (<2%), indicating good biocompatibility of L/P@Ferritin as an intravenous drug.

### L/P@Ferritin provoked ferroptosis and autophagy of TNBC cells

**Fig. 7:**
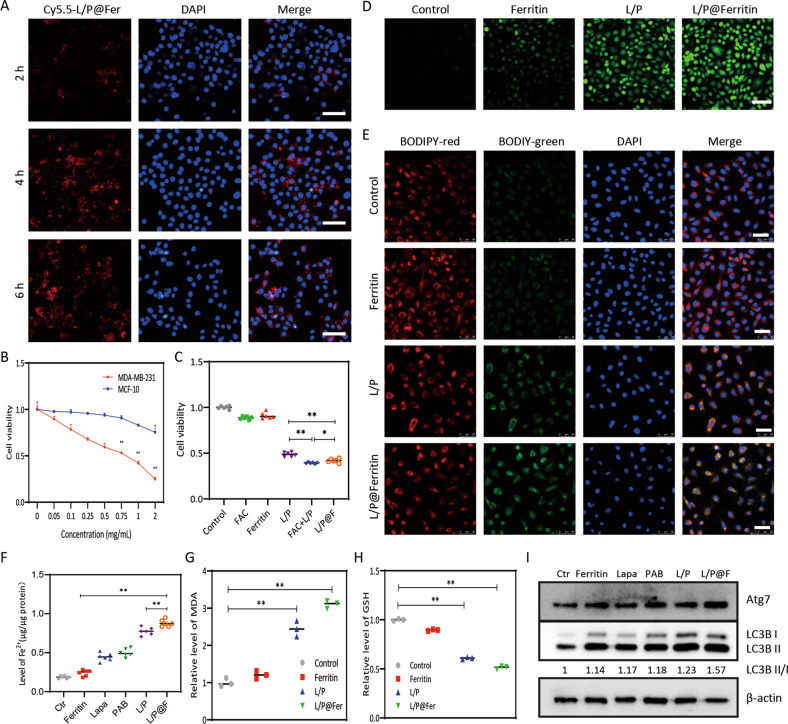
L/P@Ferritin induced ferroptosis and autophagy in vitro. **A** Fluorescence microscope images of Cy5.5-L/P@Ferritin in MDA-MB-231 cells; scale bars represent 100 μm. **B** In vitro cytotoxicity of L/P@Ferritin nanodrug in MDA-MB-231 cells and MCF-10A cells. **C** In vitro cytotoxicity of control, FAC, ferritin, lapatinib/PAB, lapatinib/PAB + LFAC, L/P@Ferritin nanodrug in MDA-MB-231 cells. **D** ROS generation detected by fluorescence microscopy when treated with L/P@Ferritin in MDA-MB-231 cells; scale bars represent 100 μm. **E** Lipid ROS generation detected by CLSM in L/P@Ferritin treated MDA-MB-231 cells; scale bars represent 50 μm. **F** Intracellular ferrous iron content in MDA-MB-231 cells was detected after different treatments. **G** Excessive MDA in MDA-MB-231 cells after treated with L/P@Ferritin. **H** Results of intracellular GSH in control, ferritin, lapatinib/PAB and L/P@Ferritin groups of MDA-MB-231 cells. **I** Autophagic proteins by western blot in MDA-MB-231 cells. *P < 0.05, **P < 0.01.

In order to verify whether L/P@Ferritin induced ferroptosis in TNBC cells and achieved the antitumor effect, we conducted relevant experiments in vitro. As shown in Fig. 7A, most of Cy5.5-L/P@Ferritin nanoparticles were accumulated in cytoplasm after incubation for 6 h. L/P@Ferritin exhibited dose-dependent cytotoxicity on MDA-MB-231 cells but had less cytotoxicity to MCF-10A cells (Fig. 7B), demonstrating selective killing ability of L/P@Ferritin on malignant tumor cells. Results of MTT experiment showed that inhibitive effect of FAC + lapatinib/PAB on MDA-MB-231 cell viability was significantly stronger than that of lapatinib/PAB, and the inhibitive effect of L/P@Ferritin nanoparticle was similar with that of FAC + lapatinib/PAB. To summarize, the iron ion in the ferritin could strengthen the killing effects of lapatinib/PAB (Fig. 7C).

Upregulate of intercellular ROS and L-ROS were by L/P@Ferritin compared to vehicle or single-agent groups verified ferroptosis induction of L/P@Ferritin (Fig. 7D, E). L/P@Ferritin significantly increased the ferrous iron levels in MDA-MB-231 cells than that of other groups (Fig. 7F). MDA levels in L/P@Ferritin-treated cells were higher than that of lapatinib/PAB (Fig. 7G); on the contrary, GSH decreased significantly after L/P@Ferritin treatment (Fig. 7H). Next, the association of L/P@Ferritin-induced tumor cell death and cellular autophagy was probed, L/P@Ferritin nanoparticles caused a more significantly increase of LC3B-II and Atg7 (Fig. 7I).

### L/P@Ferritin nanodrug inhibited tumor growth in vivo

**Fig. 8:**
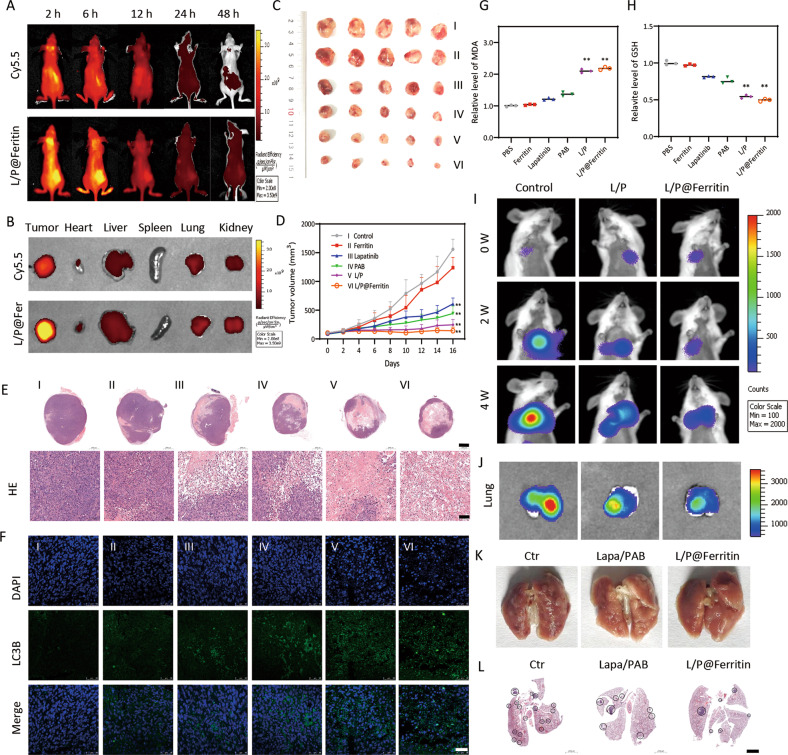
L/P@Ferritin inhibited tumor growth in vivo. **A**, **B** Fluorescence of L/P@Cy5.5-Ferritin in tumor-bearing mice at different time, and in tumor and organs which were harvested at 6 h. **C**, **D** L/P@Ferritin effectively inhibited xenograft tumor growth, I, II, III, IV, V, and VI represent the groups of PBS, ferritin, lapatinib, PAB, L/P, L/P@Ferritin. **E** HE stain of xenograft tumor, I, II, III, IV, V, and VI represent the groups of PBS, ferritin, lapatinib, PAB, L/P, L/P@Ferritin; scale bars: up panel, 2 mm; low panel, 100 μm. **F** Expression of LC3 detected by IF; scale bars represent 50 μm. **G**, **H** Overgeneration of MDA and depletion of GSH in xenograft tumor treated with L/P@Ferritin. **I**, **J** Bioluminescence images of MDA-MB-231-Luc cells in mice and extracted lungs in control, L/P and L/P@Ferritin groups respectively. **K** Numbers of pulmonary metastatic nodules in control, L/P and L/P@Ferritin groups respectively. **L** HE stain of lung in control, L/P and L/P@Ferritin groups respectively; scale bars represent 2 mm. **P < 0.01.

BALB/c nude mice with subcutaneous xenograft MDA-MB-231 tumor were used to further evaluate the lethal effects of L/P@Ferritin. In Fig. 8A, vigorous fluorescence could be monitored in the entire body at 2 h after intravenous injection of L/P@Cy5.5-Ferritin, strongest fluorescence signal accumulated in tumor at 6 h, demonstrating tumor targeting ability against TNBC tumor (Fig. 8A, B). The therapeutic effects of L/P@Ferritin were evaluated further, the volume (Fig. 8C, D) and weight (Fig. S7A) of xenograft tumors of L/P@Ferritin group were conspicuously smaller compared to the other groups at 14 days. In addition, there was no significant diversity of body weight in all experimental groups (Fig. S7B), manifesting that L/P@Ferritin had excellent security and biocompatibility. Microscopically, no substantial damages in the mouse organs (Fig. S7C). Compared to the other groups, the tumor tissues had the largest cell death range in L/P@Ferritin group (Fig. 8E). LC3B expression was augmented in xenograft tumors of L/P@Ferritin group by IF (Fig. 8F). In addition, L/P@Ferritin also resulted in increase in MDA and depletion of GSH in tumor tissues (Fig. 8G, H). In the lung metastasis model, after treated with L/P@Ferritin, tumor size was displayed by bioluminescence imaging with luciferase at different time point (Fig. 8I, J), L/P@Ferritin significantly reduced the colonization in the lungs (Fig. 8K, L).

## Discussion

Recent works demonstrated that clustered CSC phenotypes were recommended to be accountable for cancer relapse and drug resistance, consequently leading to more efficient metastasis [25]. Although previous reports evidenced that CSCs possessed unique features that rendered them sensitive to ferroptosis [26]; however, our findings hinted that MDA-MB-231 and MDA-MB-468 tumor spheres which revealed CSC-like properties were resistant to small molecular-induced ferroptotic cell death.

EGFR is frequently overexpressed in TNBC and known to promote tumor growth and metastasis [27], however, the role of EGFR in CSC clusters and polyclonal metastasis is yet to be clarified. Here, our results highlighted that sizes of TNBC tumor spheres were declined after blockade of EGFR; overexpression of EGFR increased sizes of tumor spheroid. CTC clusters were driven by aggregation of CD44^+^ CSCs [7], here, we provided evidence that CD24^low^CD44^high^ fraction clearly abrogated upon EGFR knockdown or following lapatinib treatment in TNBC cells; in addition, ALDH in TNBC cells was reduced. These findings suggested that the formation and proliferation of tumor spheres were benefited from EGFR; EGFR inhibition successfully repressed breast cancer stem cell-like population.

N-cadherin was enriched highly in invasive tumor cell lines and tumor sphere cells that regularly lacked E-cadherin [28], cell–cell junction was weakened when N-cadherin was knockdown [29]. N-cadherin induced CSC-like characteristics [30], reduction of N-cadherin led to impaired sphere formation via inactivating NF-kB [30]. Here, we found that N-cadherin was generally upregulated in spheroid breast cancer cells with exogenous expression of EGFR, blockade of EGFR in MDA-MB-231 cells gave rise to an attenuation of N-cadherin. ADH-1, an N-cadherin binding inhibitory peptide, counteracted sphere formation of EGFR-overexpressed MDA-MB-231 cells.

ECM-detached mammary epithelial cells were able to activate autophagy rapidly [31], autophagy contributed to ferroptosis by degradation of ferritin and release of ferrous iron [18, 32]. Atg5 or Atg7 silencing limited erastin-induced ferroptosis with declined lipid peroxidation and intracellular ferrous iron [33]. Here, we demonstrated that autophagosome numbers and expressions of autophagy proteins were exaggerated when MDA-MB-231 cells were treated with the combination of lapatinib and erastin, accompanied with enhanced intracellular ferrous iron. Ferritin was degraded in lapatinib and erastin co-treated MDA-MB-231 cells by proteasome; as evidenced by that an autophagy inhibitor rescued degradation of ferritin and eliminated ferroptosis.

Elevated evidence found that YAP was consistently hyperactivated in various cancer cells [34], YAP negatively regulated cell autophagy process in cancer cells, and consequently, promoted tumor progression [19, 35]. Interference of YAP considerably reinforced autophagic flux by cumulating RAC1-driven ROS, which result in deactivation of mTOR in hepatocellular carcinoma cells [36]. In this study, YAP inhibition encouraged autophagy and autophagic cell death in TNBC cells, LC3B was reduced in TNBC cell lines. Knowing that EGFR signaling pathway regulated tumor sphere formation and autophagy [5, 37], whether activation of EGFR pathway altered autophagy-associated networks was investigated. Our results exhibited that YAP was highly phosphorylated and significantly inactivated when EGFR was inhibited in MDA-MB-231 cells.

PAB is a diterpenoid acid isolated from the root bark of pseudolarix kaempferi gorden which exhibited substantial cytotoxicity and could achieve anti-cancer effects by inducing cell senescence, mitotic arrest, and autophagy [9, 22, 38]. Our results confirmed that PAB abnormally increased intracellular ferrous iron and lipid peroxidation via upregulating TfR [10] and demonstrated that PAB inhibited viabilities of TNBC cells, followed by ectopic enhancement of intracellular ferrous iron and lipid peroxidation. In addition, we validated that cotreatment with lapatinib and PAB significantly depressed cell viability in vitro, accompanied by augmented intracellular ferrous iron, overgeneration of ROS and MDA.

Ferroptosis is initiated by degradation of ferritin and consequent iron release, ferritin subunits could self-assemble into a symmetrical spherical drug nanocarrier which attracts extensive attention in cancer treatments [39]. Due to overexpression of TfR in a broad range of solid tumors; ferritin, the high targeting to TfR, may become an ideal drug carrier to stimulate ferroptosis [33, 40]. In this study, we found that TfR was upregulated by PAB which strengthened cellular uptake of Cy5.5-labeled ferritin as evidenced by accumulation and clustering of puncta in TNBC cells.

Here, ferritin-based nanotherapeutic has been developed by loading ferritin nanocages with lapatinib and PAB. The successfully prepared L/P@Ferritin nanoparticle exhibited cytotoxic effects accompanied with accumulated production of lipid peroxidation (Fig. S8). In vivo, L/P@Ferritin that aggregated within the tumor specifically confirmed by small animal imaging technology reduced the volume of xenograft tumors and numbers of lung metastases markedly. This research supplies deeper insights and a novel avenue to boost the potency of ferroptotic targeted nanomedicine against TNBC.

## Supplementary information

Supplemental data figure legends

Supplemental Fig. 1

Supplemental Fig. 2

Supplemental Fig. 3

Supplemental Fig. 4

Supplemental Fig. 5

Supplemental Fig. 6

Supplemental Fig. 7

Supplemental Fig. 8

## Data Availability

All datasets generated and analyzed during this study are included in this published article and its Supplementary Information files.
