# Supplementary material for: Co-loaded lapatinib/PAB by ferritin nanoparticles eliminated ECM-detached cluster cells via modulating EGFR in triple-negative breast cancer
Source: Cell Death Dis. 2022 Jun 20;13(6):557. doi: 10.1038/s41419-022-05007-0 (PMC9209505; doi:10.1038/s41419-022-05007-0)
Supplement: Supplementary file 1 — Supplemental data figure legends [file 41419_2022_5007_MOESM1_ESM.pdf]

Supplemental data

Figure S1. EGFR was associated with ECM-detached characteristics and CSCs ferroptosis resistance.

(A) Volcano plot showed differentially expressed genes. (B) Results of GO analyses. (C) KEGG pathway analyses. (D) Enrichment of stem cell proliferation and ferroptosis in EGFR differentially expressed genes by GSEA. (E) Overall survival analyses of different expression of EGFR in TNBC.

Figure S2. Overexpression of EGFR promoted resistance to ferroptotic cell death.

(A) EGFR-sh was constructed and significantly inhibited the expression of EGFR in MDA-MB-231 and MDA-MB-468 cells, which detected by western blot. (B) Results of MTT assay in sh-EGFR/MDA-MB-468 cells when treated with erastin. (C) Lipid peroxidation was measured using C11-BODIPY 581/591 via FCM in MDA-MB-231 cells. (D) Cell viability of EGFR-silenced MDA-MB-468 cell by Z-AVD-FMK, DFO or Fer-1. (E) Synergistic effects of lapatinib on erastin-induced growth inhibition in MDA-MB-231 cells. (F) Overexpression of EGFR by western blot in MDA-MB-453 cells. $^{**}P < 0.01$.

Figure S3. Blockage of EGFR sensitized TNBC to ferroptosis via reducing stemness.

(A) Flow cytometry was used to detect the proportion of $CD44^{high}CD24^{low}$ in MDA-MB-231 cells when treated with lapatinib. (B) ALDH subpopulation analysis in MDA-MB-231 cells. (C) Three-dimensional spheroids derived from MDA-MB-231 and MDA-MB-468 cells treated with lapatinib; scale bars represent 50 μm. (D) Colony formation assay of MDA-MB-231 and MDA-MB-468 cells treated with lapatinib. (E) Spheroid tumor cells were insensitive to ferroptosis by FCM when treated with erastin. (F) Three-dimensional spheroid formation of MDA-MB-453 cells; scale bars represent 50 μm. (G) Live/dead cell double stain; scale bars represent 200 μm.

Figure S4. EGFR inhibition promoted ferroptosis via enhancing cell autophagy.

(A) Representative confocal images of ferritin (red) and subcellular compartments (green) in the Golgi apparatus stained with GM-130 in MDA-MB-231 cells grown in the presence of 100 μg/mL FAC; scale bars represent 20 μm. (B) Partially colocalized

NCOA4 and ferritin visualized by CLSM; scale bars represent 10 μm.

Figure S5. PAB inhibited cell viability and increased intracellular iron and ROS.

(A) The effects of lapatinib and/or PAB on migration of MDA-MB-231 cells; scale bars represent 200 μm. (B, C) ROS generation in MDA-MB-231 cells by fluorescence microscopy and flow cytometry; scale bars represent 100 μm. (D) Lipid ROS generation in MDA-MB-231 cells by CLSM; scale bars represent 50 μm. (E) Lapatinib and/or PAB contributed to excessive MDA in MDA-MB-231 cells. (F) Lapatinib and PAB depleted intracellular GSH, Fer-1 and DFO rescued the GSH level. ** $P < 0.01$.

Figure S6. Synthesis and characterization of L/P@Ferritin.

(A) In vitro stability of L/P@Ferritin in water, PBS (pH 7.4) and RPMI-1640 cell culture medium. (B) In vitro stability of L/P@Ferritin in various temperature points.

Figure S7. L/P@Ferritin nanodrug inhibited of tumor growth in vivo.

(A) Tumor weight of various groups. (B) Mice body weight of various groups. (C) H&E stain of organs from various mice groups; scale bars represent 100 μm.

Figure S8. Schematic illustration.

L/P@Ferritin achieves the purpose of better delivery of lapatinib and PAB to weak stemness, induce autophagy and ferroptosis.
