# Supplementary figures and images for: Co-loaded lapatinib/PAB by ferritin nanoparticles eliminated ECM-detached cluster cells via modulating EGFR in triple-negative breast cancer
Source: Cell Death Dis. 2022 Jun 20;13(6):557. doi: 10.1038/s41419-022-05007-0 (PMC9209505; doi:10.1038/s41419-022-05007-0)

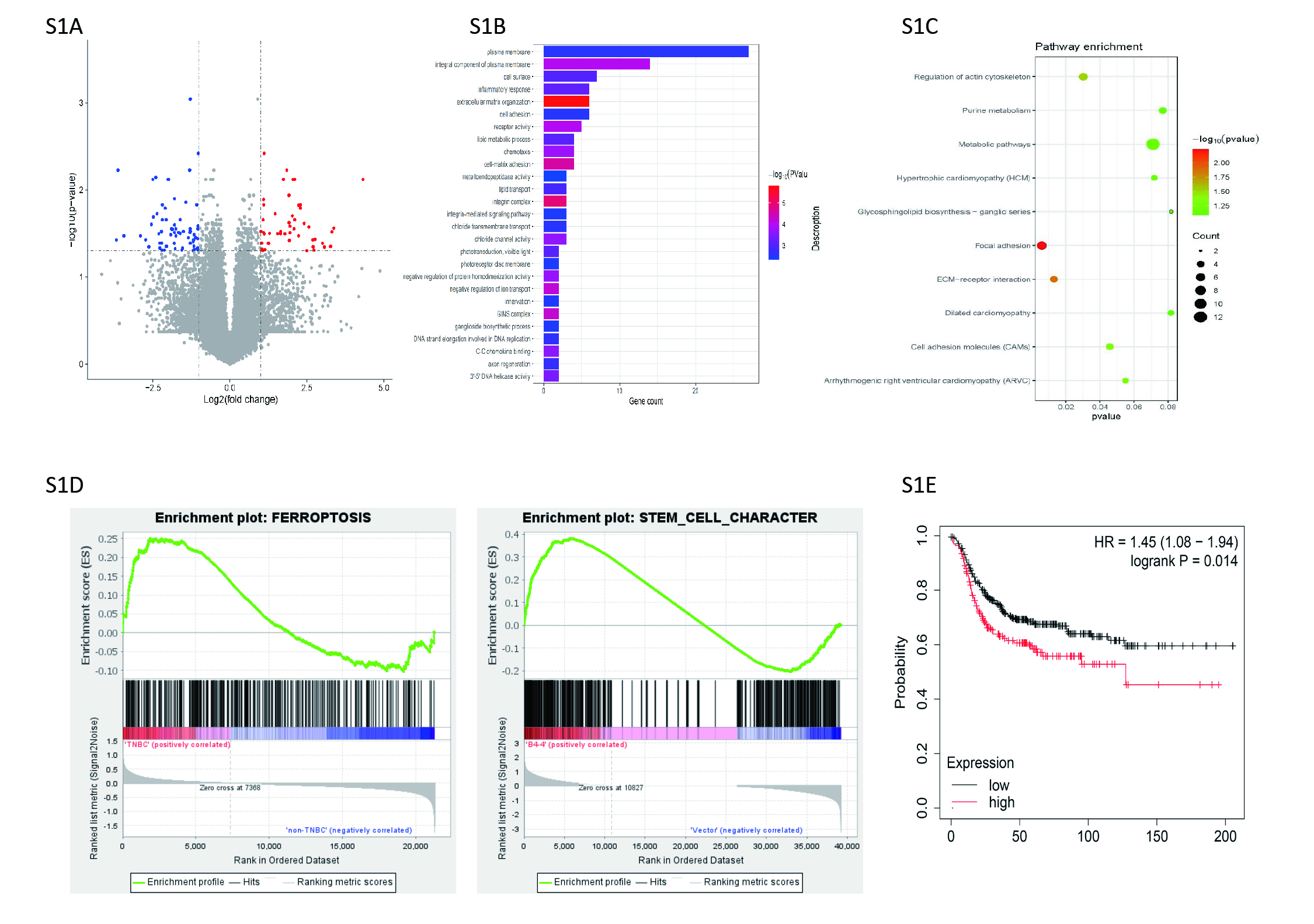

Supplement: Supplementary file 2 — Supplemental Fig. 1 [file 41419_2022_5007_MOESM2_ESM.jpg]

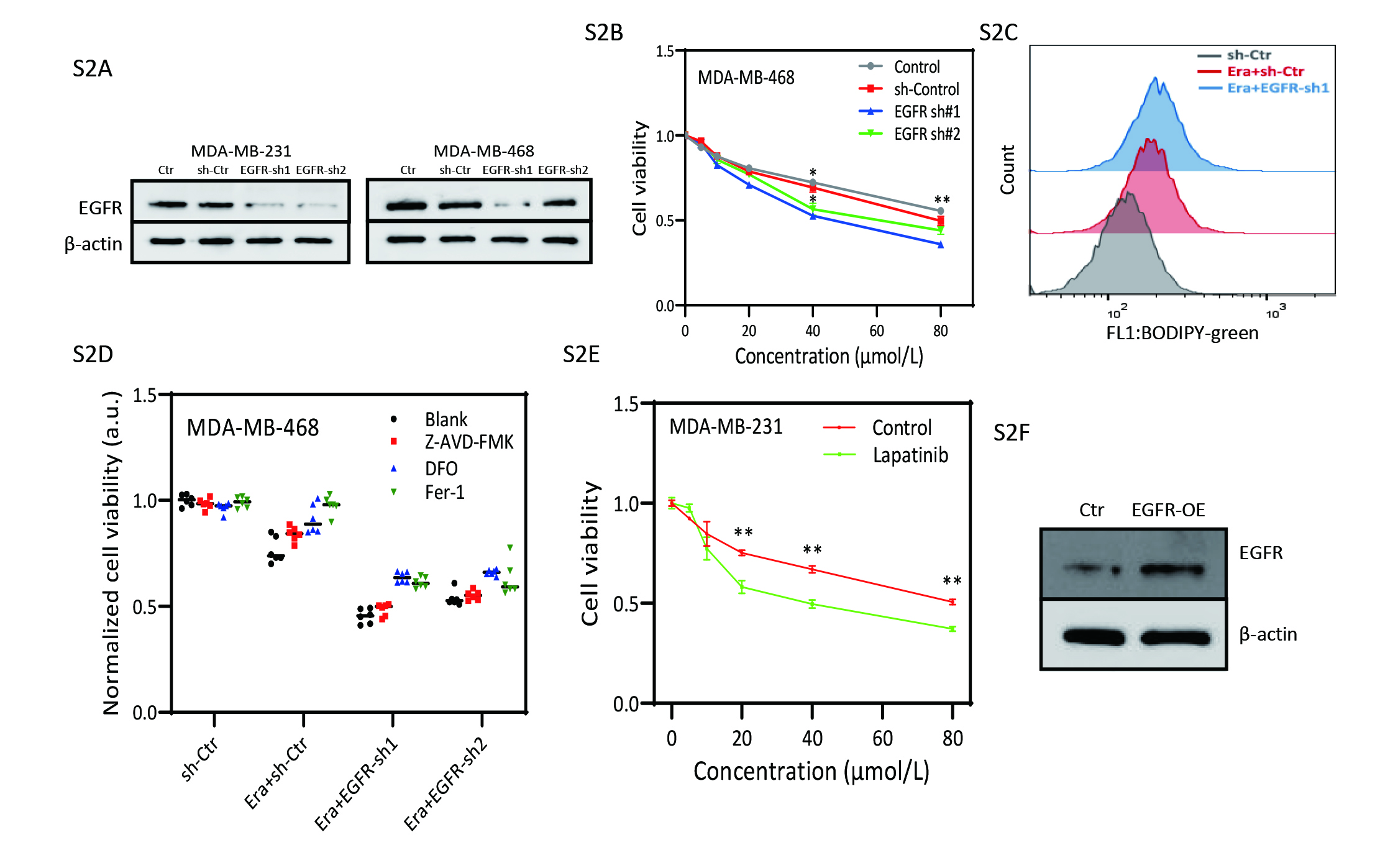

Supplement: Supplementary file 3 — Supplemental Fig. 2 [file 41419_2022_5007_MOESM3_ESM.jpg]

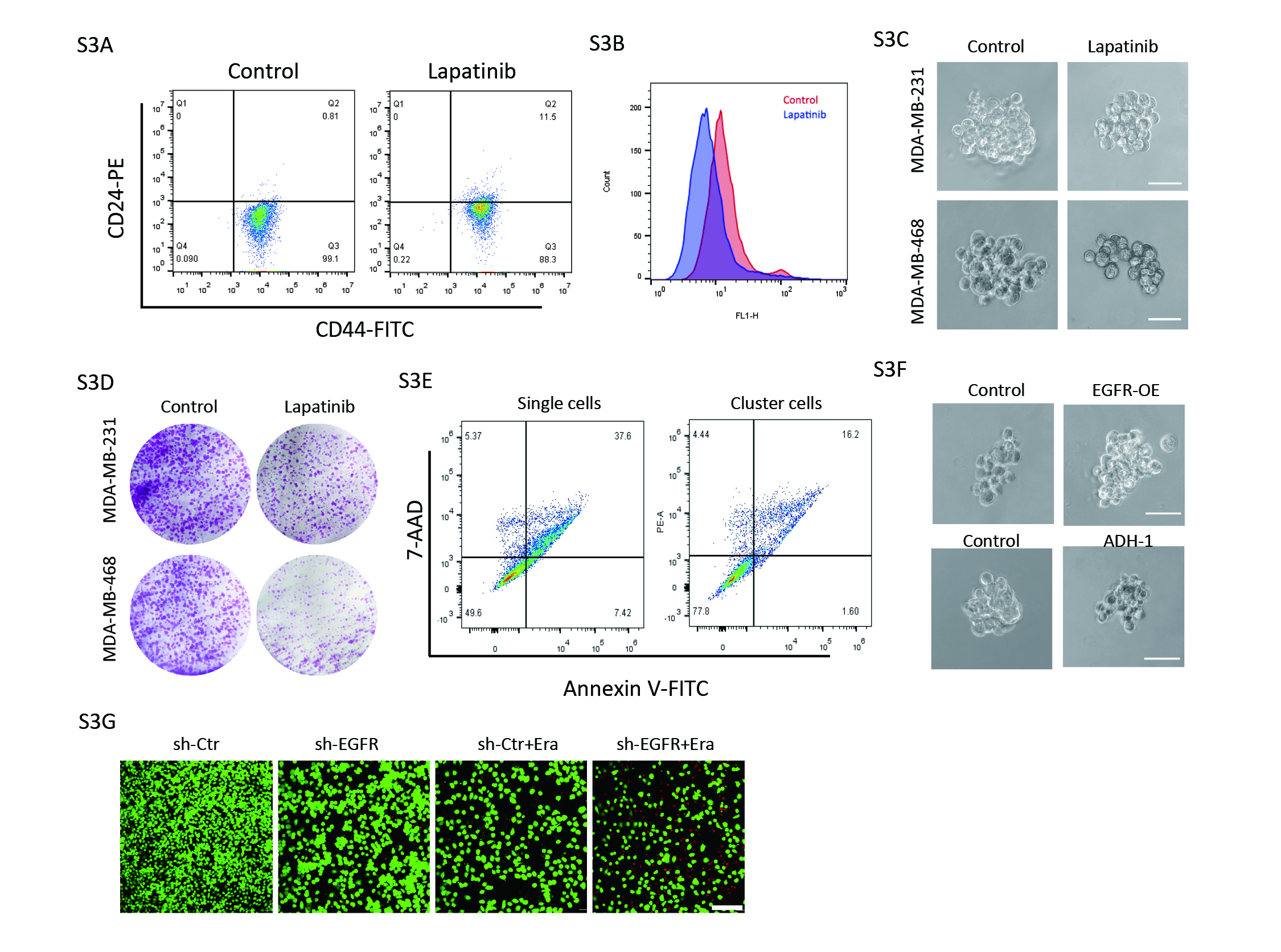

Supplement: Supplementary file 4 — Supplemental Fig. 3 [file 41419_2022_5007_MOESM4_ESM.jpg]

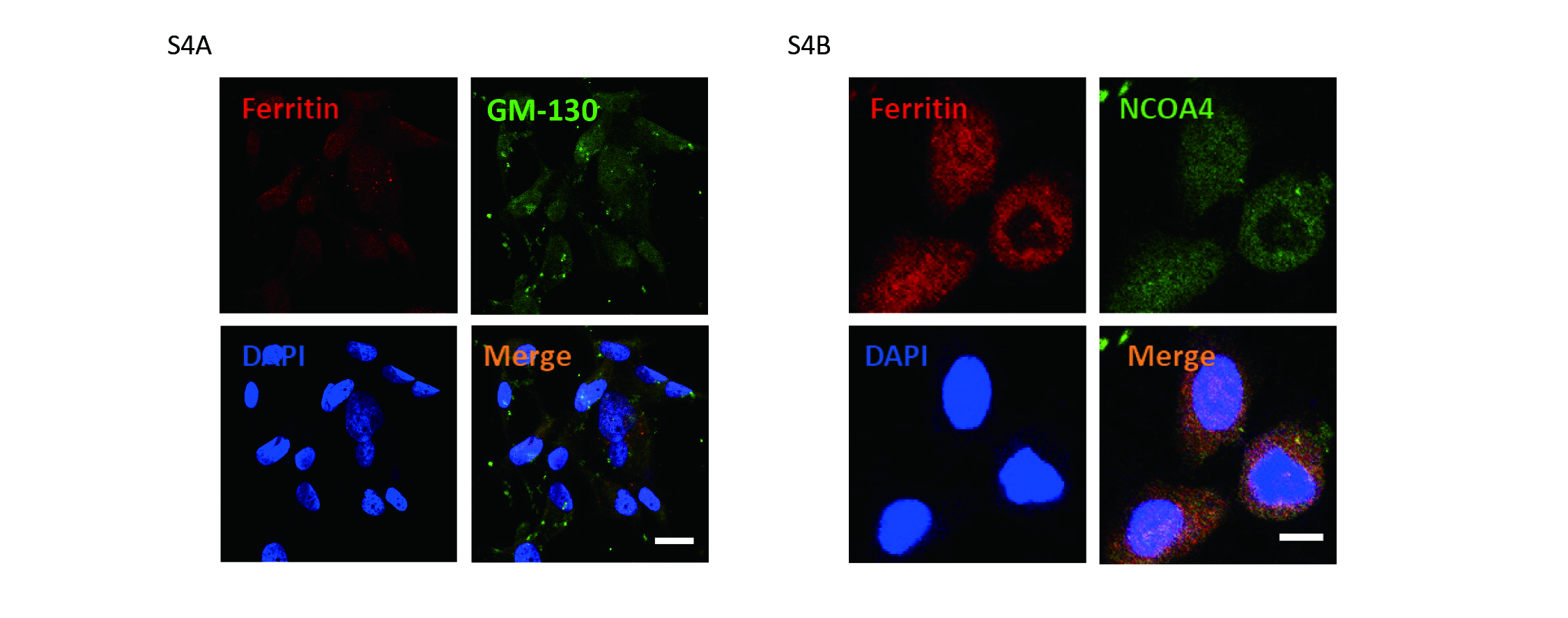

Supplement: Supplementary file 5 — Supplemental Fig. 4 [file 41419_2022_5007_MOESM5_ESM.jpg]

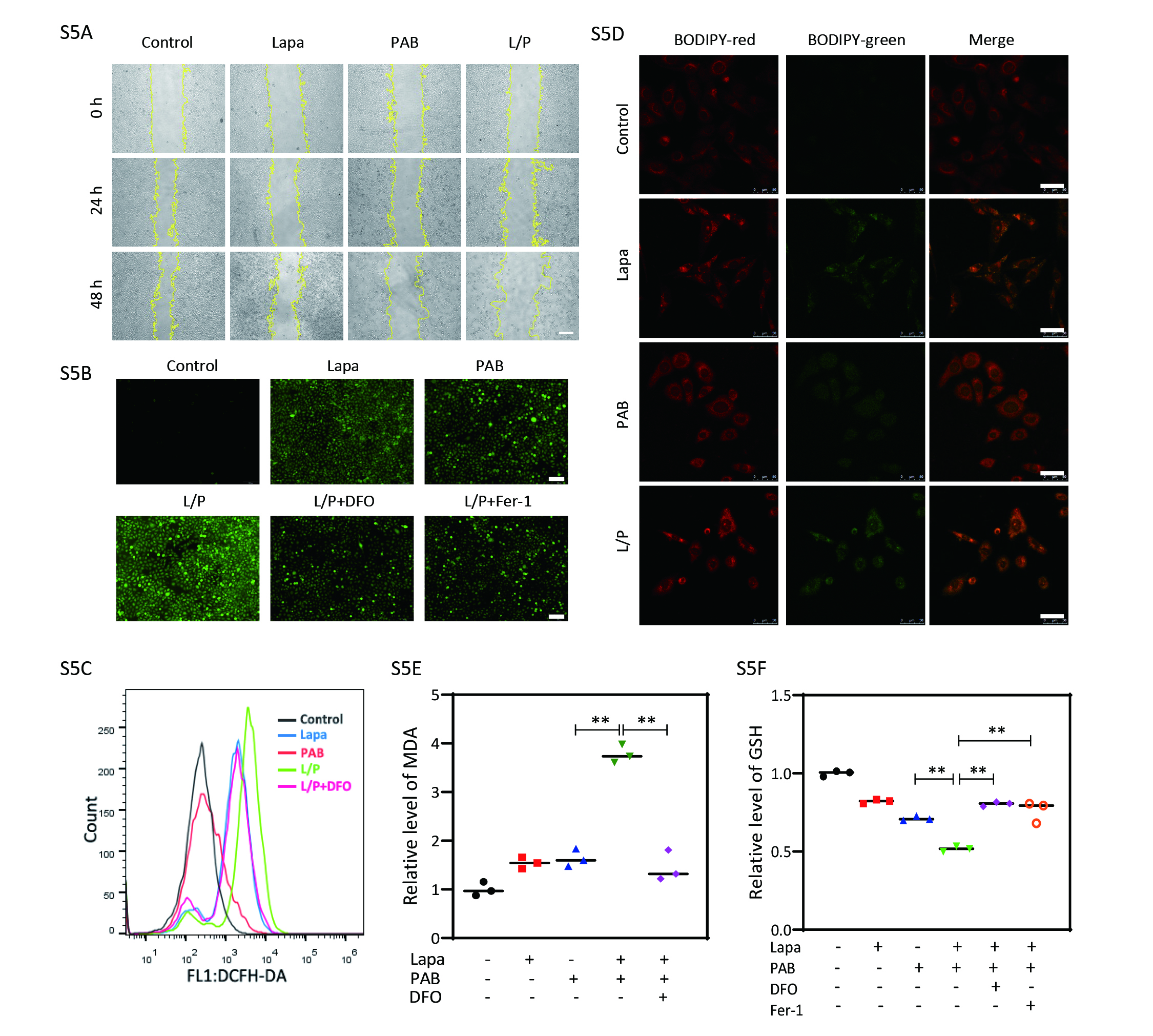

Supplement: Supplementary file 6 — Supplemental Fig. 5 [file 41419_2022_5007_MOESM6_ESM.jpg]

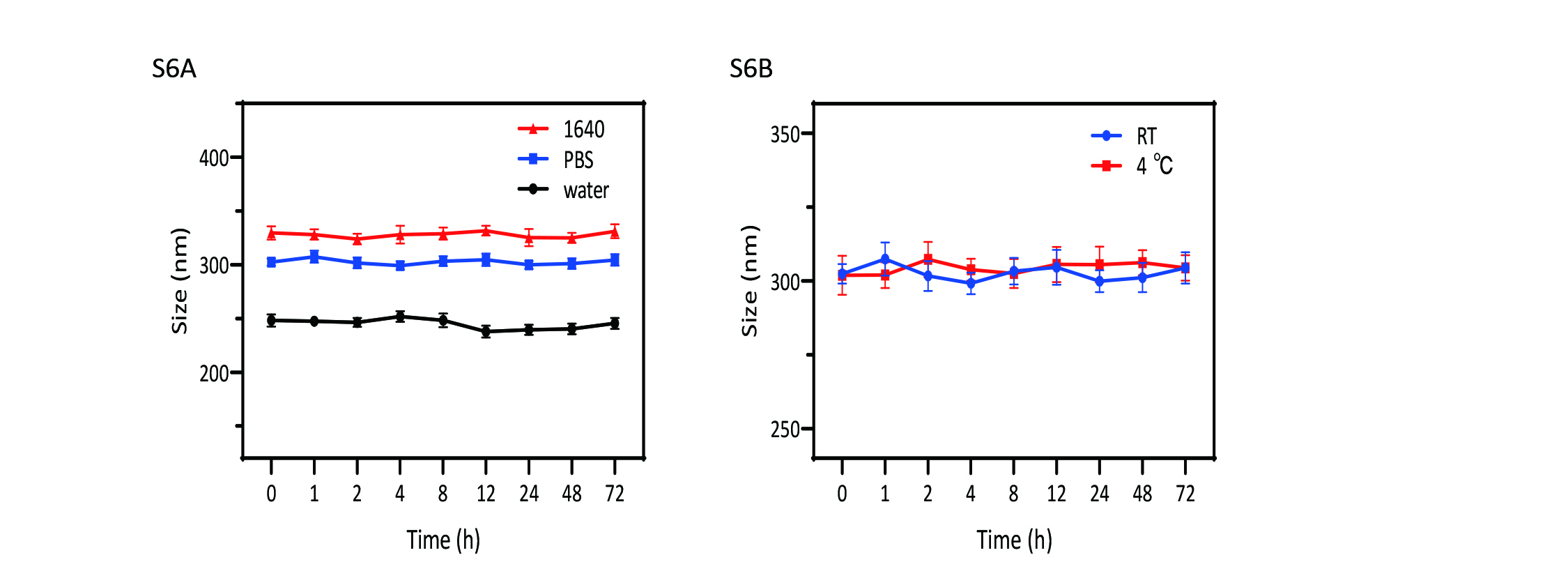

Supplement: Supplementary file 7 — Supplemental Fig. 6 [file 41419_2022_5007_MOESM7_ESM.jpg]

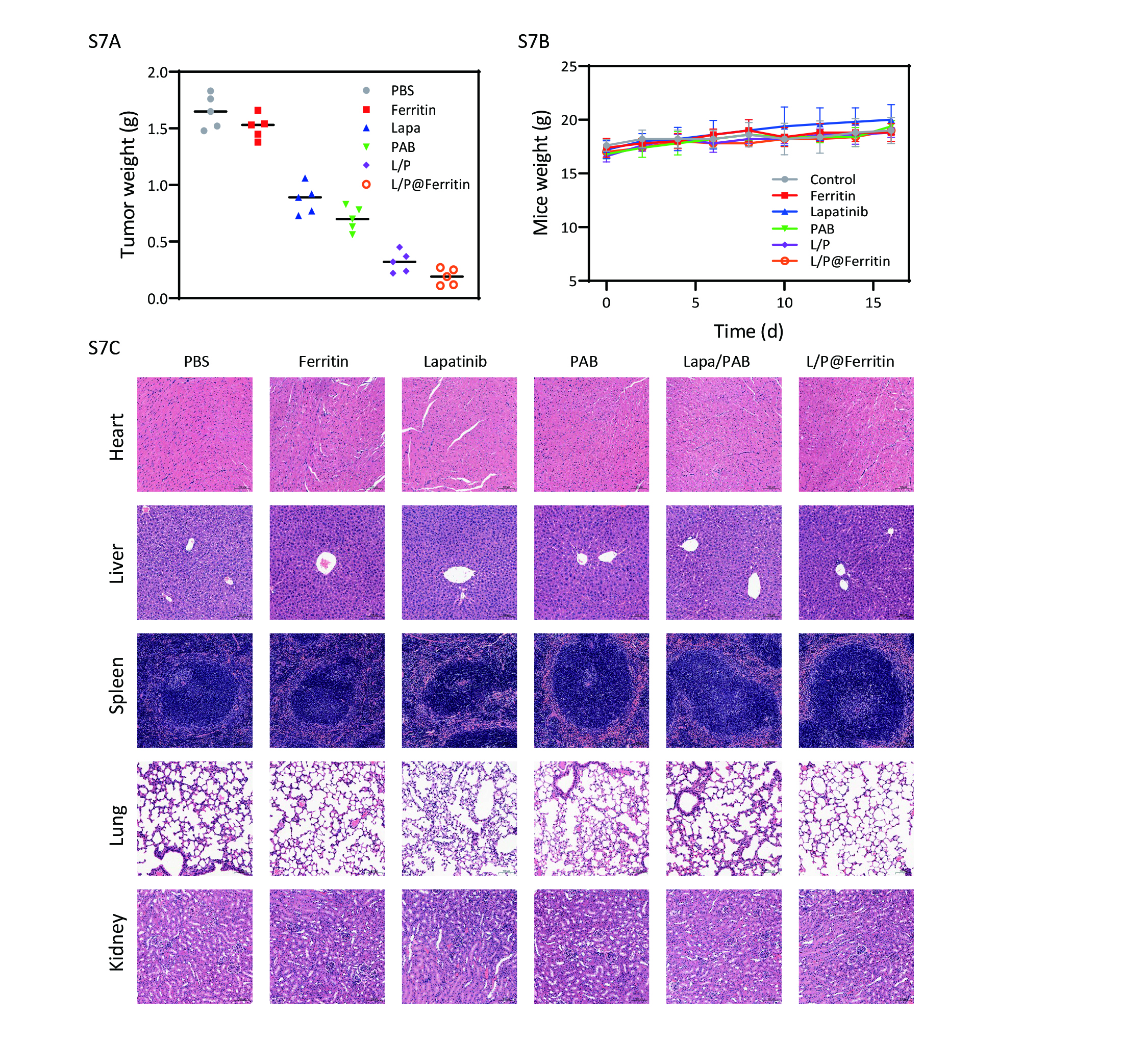

Supplement: Supplementary file 8 — Supplemental Fig. 7 [file 41419_2022_5007_MOESM8_ESM.jpg]

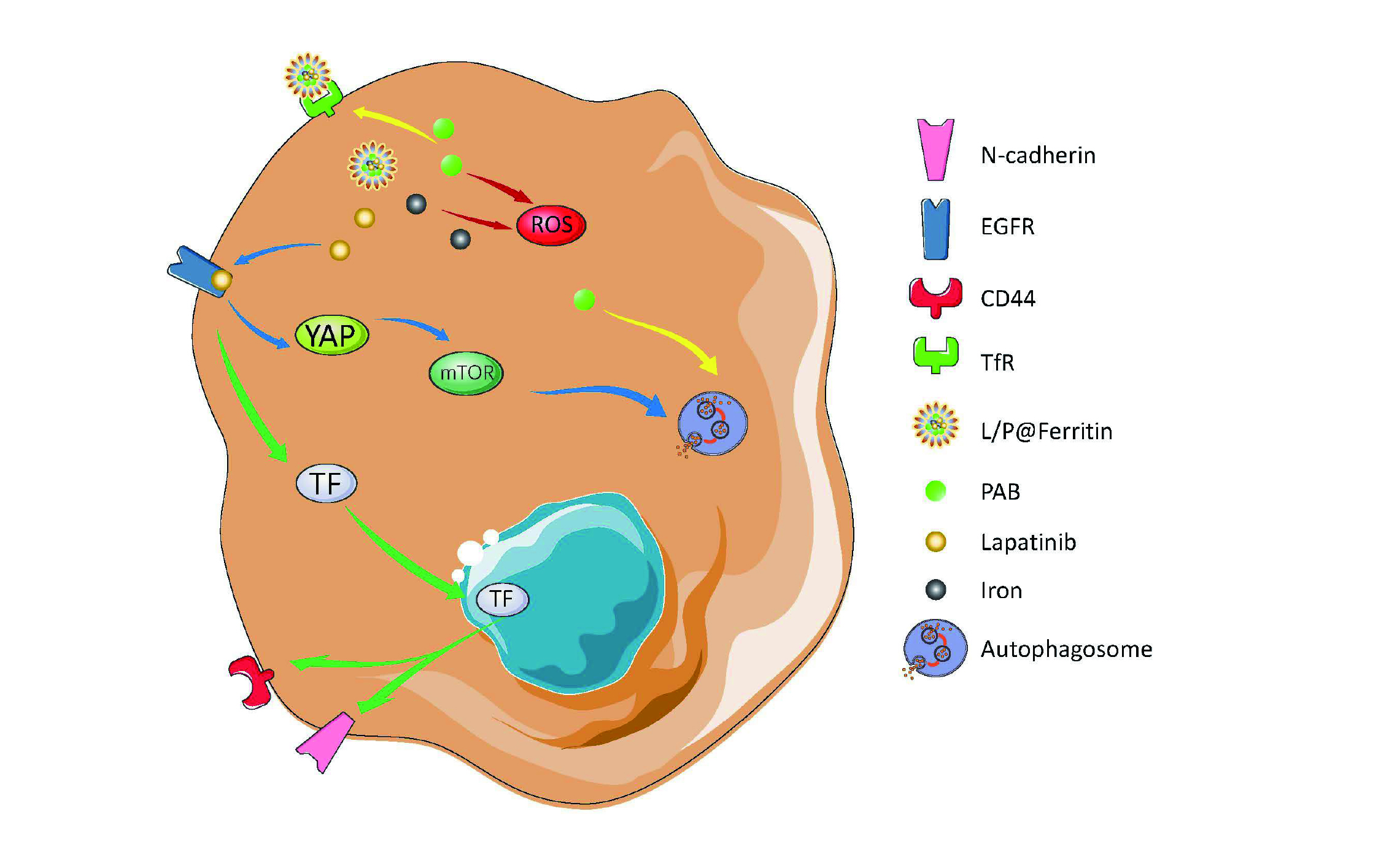

Supplement: Supplementary file 9 — Supplemental Fig. 8 [file 41419_2022_5007_MOESM9_ESM.jpg]
